# Multiply restimulated human cord blood-derived Tregs maintain stabilized phenotype and suppressive function and predict their therapeutic effects on autoimmune diabetes

**DOI:** 10.1186/s13098-024-01277-0

**Published:** 2024-03-21

**Authors:** Yuanjie Bi, Ran Kong, Yani Peng, Donghua Cai, Yu Zhang, Fan Yang, Xia Li, Wen Deng, Fang Liu, Binbin He, Chuqing Cao, Chao Deng, Xiaohan Tang, Li Fan, Haibo Yu, Zhiguang Zhou

**Affiliations:** 1 National Clinical Research Center for Metabolic Diseases, Key Laboratory of Diabetes Immunology, Ministry of Education, Hunan Engineering Research Center of Cell Therapy for Diabetes, and Department of Metabolism and Endocrinology, The Second Xiangya Hospital of Central South University, Changsha, China; 2 Department of Obstetrics and Gynecology, The Second Xiangya Hospital of Central South University, Changsha, China

**Keywords:** Regulatory T cells, Umbilical cord blood, Cell proliferation, Autoimmune diabetes, Cell therapy

## Abstract

**Background:**

Regulatory T cells (Tregs) are involved in the maintenance of immune homeostasis and immune regulation. Clinical trials on the adoptive transfer of Tregs have been ongoing for > 10 years. However, many unresolved issues remain in the production of readymade Treg products and selection of patients. Hence, this study aimed to develop a method to expand off-the-shelf Tregs derived from umbilical cord blood (UCB-Tregs) in vitro without changing their phenotype and inhibitory function. In addition, the study intended to design an approach to precisely select patients who are more likely to benefit from the adoptive Treg transfer therapy.

**Methods:**

UCB-Tregs were isolated and cultured in a medium containing human recombinant IL-2 and rapamycin and then multiply restimulated with human T-activator CD3/CD28 dynabeads. The phenotype and suppressive capacity of Tregs were assessed on days 18 and 42. The relationship between the suppressive function of UCB-Tregs in vitro and clinical indicators was analyzed, and the ability of the in vitro suppressive capacity to predict the in vivo therapeutic effects was evaluated.

**Results:**

UCB-Tregs expanded 123-fold and 5,981-fold at 18 and 42 days, respectively. The suppressive function of UCB-Tregs on the proliferation of immune cells at 42 days was not significantly different compared with that of UCB-Tregs obtained at 18 days. The suppression rate of UCB-Tregs to PBMCs was negatively correlated with the course of diabetes. Moreover, the high-suppression group exhibited a better treatment response than the low-suppression group during the 12-month follow-up period.

**Conclusions:**

Multiply restimulated UCB-Tregs expanded at a large scale without any alterations in their classical phenotypic features and inhibitory functions. The suppressive function of Tregs in vitro was negatively correlated with the disease duration. The present study revealed the possibility of predicting the in vivo therapeutic effects via the in vitro inhibition assay. Thus, these findings provided a method to obtain off-the-shelf Treg products and facilitated the selection of patients who are likely to respond to the treatment, thereby moving toward the goal of precision treatment.

## Background

Autoimmune diabetes is a chronic disease caused by the autoimmune destruction of insulin-producing pancreatic β cells that leads to insulin dependence for survival. While exogenous insulin is not a cure, efforts are underway to reverse autoimmunity during diabetes using immunomodulatory therapies. One of such promising therapies is regulatory T cell (Treg)-based cell therapy [1, 2]. Tregs have been shown to be decreased and/or functionally impaired in autoimmune diabetes [3–6]. Moreover, animal studies have demonstrated the therapeutic potential of Tregs in autoimmune diabetes [7–12]. Several studies have evaluated noncell immunotherapy on various pathogenic targets of autoimmune diabetes [13–16]. In particular, the anti-CD3 antibody teplizumab has exhibited promising results [17, 18]. Based on the outstanding outcomes in clinical trials, it was approved by the US Food and Drug Administration in 2022 to delay the onset of stage 3 type 1 diabetes (T1D) in patients aged > 8 years and in adults with T1D at stage 2. It is worth noting that some non-Treg therapies in autoimmune diabetes may work by affecting the percentage of Tregs or the ratio of Tregs to other immune cells [16, 19–21].

At present, only two clinical trials on autologous peripheral blood-derived ex vivo polyclonally expanded Tregs alone for treating autoimmune diabetes have been published [22–24]. Although the safety and efficacy of the therapy have been tentatively demonstrated, because of the small sample size, additional proof of its exact efficacy is needed. Moreover, at present, concerns remain regarding the contamination of Tregs with effector T cells along with the functional stability of Tregs (1). Furthermore, the current method does not produce ready-to-use products and is not convenient for repeated treatment cycles. In this context, umbilical cord blood-derived Tregs (UCB-Tregs) may provide an attractive alternative source for Treg products owing to their several unique characteristics, including easier sorting through the expression of CD25 [25–27], more CD45RA^+^ immature cells [26, 28], better maintenance of the stability of phenotype and function during in vitro expansion, and no effector T cell contamination [29–31], among others. However, no studies have been published on UCB-Tregs for autoimmune diabetes, and there is a lack of treatment efficacy predictors, which are important for precision medicine. These limitations encouraged us to perform large-scale ex vivo amplification experiments using UCB-Tregs for clinical intervention in patients with autoimmune diabetes. Herein, we report a method for the rapid sorting and large-scale expansion of UCB-Tregs in vitro with the maintenance of their phenotypic and functional stability in addition to a preliminary analysis of indicators predicting the therapeutic effect in treated patients.

## Methods

### UCB-Treg isolation and ex vivo expansion

Human UCB units derived from healthy donors were provided by the Department of Obstetrics and Gynecology at the Second Xiangya Hospital of Central South University (Changsha, Hunan, China). All enrolled donors signed an informed consent form. The potential presence of common infectious diseases was assessed in all UCB units, as requested by the regulatory agencies. Only the UCB units fulfilling the release criteria were used to prepare UCB-derived mononuclear cells (UBMCs) for the study. UBMCs were isolated using Histopaque-1077 (Sigma-Aldrich, St. Louis, MO, USA). Thereafter, EasyStep Human CD4^+^CD127^low^CD25^+^ Regulatory T Cell Isolation Kit (StemCell Technologies, Vancouver, BC, Canada) was used to isolate Tregs from frozen or fresh UBMCs. Briefly, CD25^+^ cells were isolated via column-free immunomagnetic positive selection. Then, non-CD4^+^ and CD127^high^ cells were depleted, followed by the removal of the bound magnetic particles from the EasySep™-isolated CD25^+^ cells. Finally, the unwanted non-Tregs were targeted for depletion.

Tregs were cultured in 48-well flat-bottom plates (Costar, Cambridge, MA) at a concentration of 10^6^ cells/mL in X-VIVO 15 medium (Lonza Group Ltd, Basel, Switzerland) supplemented with 5% human AB serum (Gemini Bio-Products, Sacramento, CA), 100 nM rapamycin [32, 33] (BioVision, Milpitas, CA), 2 mM L-glutamine (Sigma, St. Louis, MO), 0.025 M HEPES (Hyclone, South Logan, UT), 5 mM sodium pyruvate (Sigma-Aldrich, St. Louis, MO), 1% nonessential amino acids (Sigma-Aldrich, St. Louis, MO), and 0.5% penicillin-streptomycin (Gibco/Invitrogen, Grand Island, NY) and incubated at 37 °C with 5% CO_2_. Tregs were activated using Dynabeads Human T-Activator CD3/CD28 (Thermo Fisher Scientific, Waltham, MA) at a 1:1 bead:cell ratio. Human recombinant IL-2 (400 IU/mL; R&D Systems, Minneapolis, MN) and the culture medium were added on day 2 to make up the final culture volume of 1 ml. Fresh medium and IL-2 were added every third day. Anti-CD3/CD28 beads were removed every 10 days and Tregs were restimulated with new beads at a 1:1 ratio. Cell count was performed while changing beads, and on days 18 and 42.

### UCB-Treg immunophenotyping identification

The phenotype of Tregs was evaluated using flow cytometry (DxP Athena™, Cytek Biosciences). Freshly isolated Tregs as well as Tregs expanded for 18 and 42 days were collected for phenotype identification. Dead cells were removed based on the Zombie Aqua™ Fixable Viability Kit (BioLegend, San Diego, CA, USA) staining. Nonspecific Fc receptors were blocked using Human BD Fc Block (Becton Dickinson Biosciences, Franklin lakes, NJ, USA), and the cells were incubated with different mouse anti-human monoclonal antibodies (mAb), including APC-conjugated anti-CD3 (UCHT1), PerCP-Cy5.5-conjugated anti-CD4 (RPA-T4), APC-Cy7-conjugated anti-CD8 (SK1), BB515-conjugated anti-CD25 (2A3), and PE-conjugated anti-CD127 (HIL-7R-M21). The following isotype control antibodies were used: APC mouse IgG1 (MOPC-21), PerCP-Cy5.5 mouse IgG1 (MOPC-21), APC-Cy7 mouse IgG1 (MOPC-21), BB515 mouse IgG1 (X40), and PE mouse IgG1 (MOPC-21). All antibodies were from Becton Dickinson Biosciences unless otherwise specified, and the staining was performed according to the manufacturer’s instructions.

For the detection of functional markers of Tregs, APC-conjugated anti-Foxp3 (PCH101) and Foxp3/transcription factor staining buffer set were purchased from Invitrogen, BV421-conjugated mouse anti-human CD152 (cytotoxic T lymphocyte-associated antigen 4, CTLA-4) antibody (BNI3) was bought from Becton Dickinson Biosciences, and PerCP/Cyanine5.5-conjugated mouse anti-human CD357 (glucocorticoid-induced tumor necrosis factor receptor-related protein, GITR) antibody (108 − 17) was obtained from BioLegend. Staining was performed according to the manufacturer’s instructions. The fluorescence minus one (FMO) control [34], which included all of the antibody conjugates present in the test samples except one, was used to gate Foxp3, CTLA-4, and GITR.

Flow cytometric data were analyzed using the FlowJo X software (Tree Star, Ashland, OR). All Treg products passed the following lot release criteria [35]: purity of CD4^+^CD25^+^CD127^−^ ≥ 60%, purity of CD3^+^CD8^+^cells < 10%, and endotoxin < 5 EU/kg.

### Suppressive capacity assessment of UCB-Tregs

The suppressive capacity of Tregs was assessed on days 18 and 42 using carboxyfluorescein diacetate succinimidyl ester (CFSE, Thermo Fisher Scientific, Waltham, MA, USA) for defining the dividing cells. PBMCs from patients with autoimmune diabetes were stained with CFSE according to the manufacturer’s protocol and then cocultured with various ratios of Tregs (1:0, 1:1, 2:1, 4:1, and 8:1) with or without anti-CD3/CD28 beads for 72 h (h). Then, the cells were collected and stained with several antibodies, including live/dead fluorescent dye, APC-Cy7-conjugated anti-CD8 (SK1), and PerCP-Cy5.5-conjugated anti-CD4 (RPA-T4). Flow cytometric data were analyzed using the FlowJo X software (Tree Star, Ashland, OR). Dead cells were excluded as mentioned above, and PBMCs were distinguished from Tregs via CFSE staining. The suppression rate was calculated as follows: (% of proliferating PBMCs or CD4^+^ T cells or CD8^+^ T cells cocultured without Tregs -% of proliferating PBMCs or CD4^+^ T cells or CD8^+^ T cells with Tregs) / (% of proliferating PBMCs or CD4^+^ T cells or CD8^+^ T cells cocultured without Tregs).

### Patients and follow-up

Participants aged between 6 and 60 years who were diagnosed with autoimmune diabetes according to the American Diabetes Association standard criteria within 2 years; were positive for at least one autoantibody to islet cells such as glutamic acid decarboxylase antibody (GADA), protein-tyrosine phosphatase antibody (IA-2 A), or zinc transport 8 antibody (ZnT8A); and had a peak C-peptide level of at least 200 pmol/L during a mixed meal tolerance test (MMTT) were eligible for participation in the trial. PBMCs from nine patients with autoimmune diabetes were cocultured with 18-day UCB-Tregs before their introduction in patients included in the clinical trial (Safety Study and Therapeutic Effects of Umbilical Cord Blood Treg on Autoimmune Diabetes, ClinicalTrials.gov no. NCT02932826; final data of this trial have not yet been published). They were also used for the subsequent retrospective analysis of the factors affecting the therapeutic efficacy. Whole blood was collected to isolate PBMCs before UCB-Treg infusion, followed by their coculture with UCB-Tregs. The flow cytometric data were analyzed as mentioned above. Blood samples were also collected for the assessment of fasting blood glucose (FBG), postprandial blood glucose (PBG), fasting C-peptide (FCP), postprandial C-peptide (PCP), hemoglobin A1c (HbA1c), and MMTT at the baseline, month 1, month 3, month 6, month 9, and month 12 of the follow-up period to evaluate the efficacy of the therapy. The trapezoid rule was used to calculate the area under the curve (AUC) for C-peptide over 2 h of MMTT (C-peptide AUC). The trial was approved by the Ethics Committee of the Second Xiangya Hospital, Central South University (IRB number No. 021 of 2016). Written informed consent was obtained from all the enrolled patients.

### Statistical analysis

Statistical analysis was performed using SPSS 26.0 software (SPSS, Chicago, IL) and GraphPad Prism 9 software (GraphPad Software, San Diego, CA, USA). Assessment for normal distribution was performed using the Shapiro–Wilk test. Independent Student’s *t*-test or Mann-Whitney U test (nonparametric test) was performed for two-group comparison, whereas one-way ANOVA followed by Tukey’s post hoc test or Kruskal-Wallis test (nonparametric test) followed by Dunn’s multiple comparison post-test was used for comparing three or more groups according to the normality of the data. Correlations between the suppression rate and clinical data were determined using Pearson or Spearman correlation analysis. Generalized estimating equations (GEEs) were used for the repeated measurement data of patients during the follow-up from the baseline to 12 months. The group–time interaction was considered in the model. Results are expressed as the mean ± standard deviation unless otherwise stated. A value of *p* < 0.05 was considered statistically significant.

## Results

### Fold expansion and purity of UCB-Tregs expanded in vitro

**Fig. 1:**
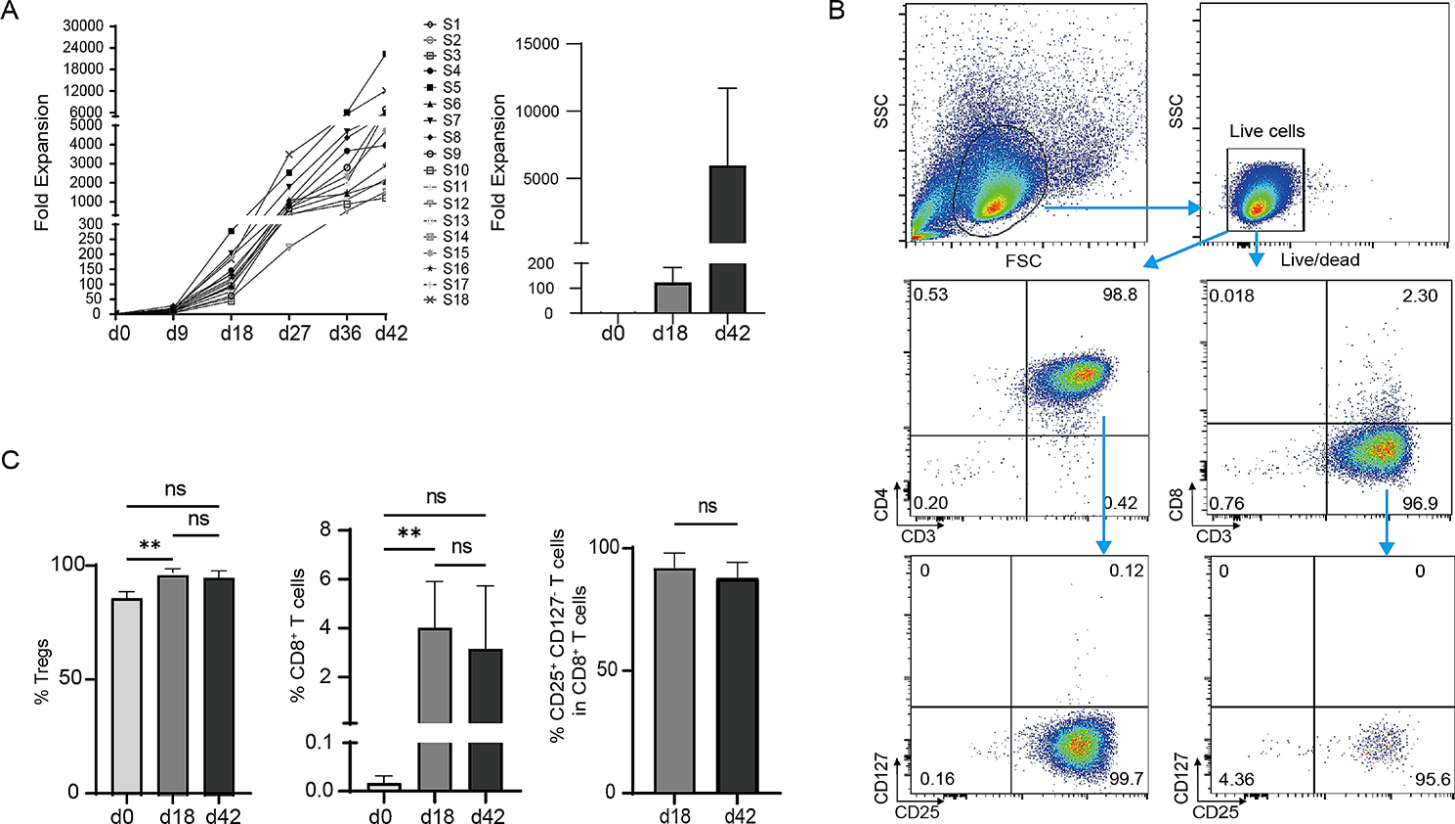
Fold expansion and purity of UCB-Tregs expanded for 18 and 42 days in vitro. (A) Fold expansion; n = 18 (d18), n = 13 (d42). (B) Representative images showing gating strategy for the immunophenotyping of Tregs stained with CD3, CD4, CD25, CD127, and CD8. (C) Percentage of Tregs (CD3+CD4+CD25+CD127− cells); n = 3 (d0), n = 15 (d18), and n = 13 (d42) and CD8+ T cells (CD3+CD8+ cells); n = 3 (d0), n = 15 (d18), and n = 13 (d42). Percentage of CD25+CD127− T cells in CD8+ T cells; n = 15 (d18), n = 13 (d42). Purity among d0, d18, and d42 Tregs was compared using the Kruskal–Wallis test, followed by Dunn’s multiple comparison test. Comparison of percentage of CD25+CD127− T cells in CD8+ T cells was analyzed using Mann-Whitney test. **p < 0.01, ns indicates no significant difference

UCB-Tregs from 18 healthy donors were sorted and cultured in vitro for 18 days, whereas 13 of them were expanded to 42 days. An expansion of 123 ± 60-fold and 5,981 ± 5,721-fold was obtained over a period of 18 and 42 days, respectively. The fold expansion data of UCB-Tregs from healthy donors are shown in Fig. 1A. The purity of UCB-Tregs (CD3^+^CD4^+^CD25^+^CD127^−^ T cells) using the surface markers CD3, CD4, CD25, CD127, and CD8 is shown in Fig. 1B. The UCB-Treg percentage in the 18-day culture (97.13 ± 1.54%) was higher than that in freshly isolated Tregs (87.00 ± 2.92%), and the phenotype remained stable even after 42 days of expansion (96.00 ± 3.01%) (Fig. 1C). Although the percentage of CD8^+^ T cells expanded for 18 and 42 days was higher than that of freshly isolated cells, it did not exceed 8% (Fig. 1C) and most of them were CD8^+^CD25^+^CD127^−^ T cells (Fig. 1C), which had been characterized as CD8^+^ Tregs by Churlaud et al. [36].

### Expression of Foxp3, CTLA-4, and GITR

**Fig. 2:**
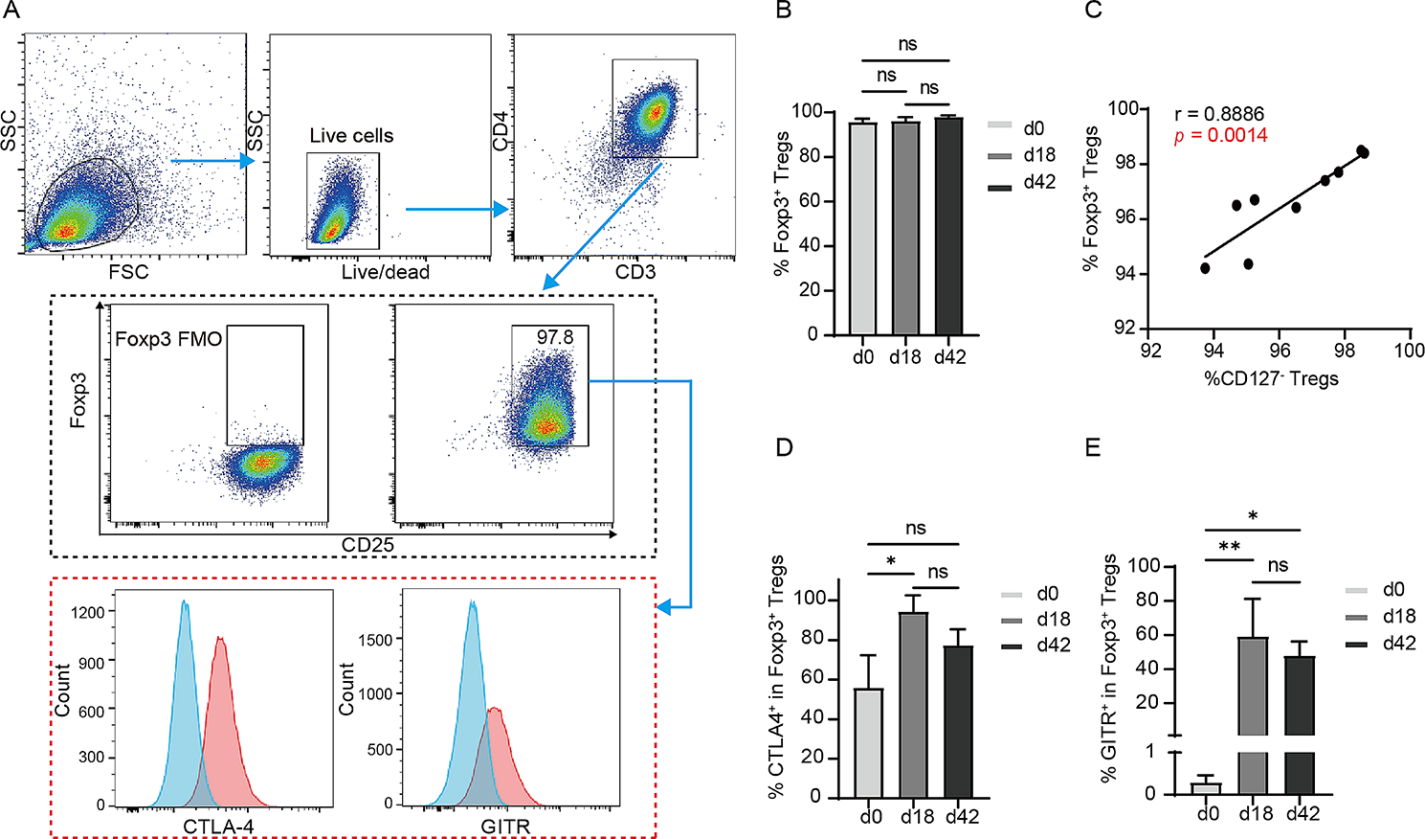
Expression of Foxp3 in sorted or expanded cells and that of CTLA-4 and GITR on CD4+CD25+Foxp3+ Tregs. (A) Representative gating strategy of Foxp3, CTLA-4, and GITR expression. Cells were identified using their forward- (FSC) and side-scatter (SSC) properties. Live cells were gated using Zombie Aqua™ Fixable Viability Kit staining and then examined for the coexpression of CD3 and CD4. Foxp3 expression in CD4+CD25+ T cells is plotted on the basis of Foxp3 fluorescence minus one (FMO). CTLA-4 and GITR expression (red histogram) on CD4+CD25+Foxp3+ Tregs were identified via the corresponding FMO (blue histogram). (B) Percentage of CD4+CD25+ Foxp3+ Tregs expanded for 0, 18, and 42 days (n = 3, each group). (C) Correlation between the percentage of CD4+CD25+CD127− Tregs and CD4+CD25+ Foxp3+ Tregs (n = 9). (D) Percentage of CTLA-4+ cells in CD4+CD25+Foxp3+ Tregs expanded for 0, 18, and 42 days (n = 3, each group). (E) Percentage of GITR+ cells in CD4+CD25+ Foxp3+ Tregs expanded for 0, 18, and 42 days (n = 3, each group). P-values for differences between groups were determined using one-way ANOVA, followed by Tukey’s multiple comparisons test in (B), (D), and (E). Pearson correlation analysis was used in (C). *p < 0.05, **p < 0.01, ns indicates no significant difference

Considering the key roles of Foxp3, CTLA-4, and GITR in Tregs [37, 38], we detected their expression by analyzing the data from flow cytometry in freshly sorted UCB-Tregs, which were also expanded for 18 and 42 days (Fig. 2A). The expression of Foxp3 in Tregs was high in both freshly sorted cells (95.81 ± 1.38%) and in those expanded for 18 (96.17 ± 1.68%) or 42 days (98.10 ± 0.61%) (Fig. 2B). We also found that CD127^−^ Tregs (CD3^+^CD4^+^CD25^+^CD127^−^ T cells) and Foxp3^+^ Tregs (CD3^+^CD4^+^CD25^+^Foxp3^+^ T cells) were linearly correlated (Fig. 2C, *r* = 0.8886, *p* = 0.0014), indicating that the low expression of CD127 was similar to the high expression of Foxp3 in distinguishing Tregs. In addition, the surface expression of CTLA-4 (Fig. 2D) and GITR (Fig. 2E) on Foxp3^+^ Tregs was upregulated after expansion.

### Suppressive function of UCB-Tregs expanded in vitro

**Fig. 3:**
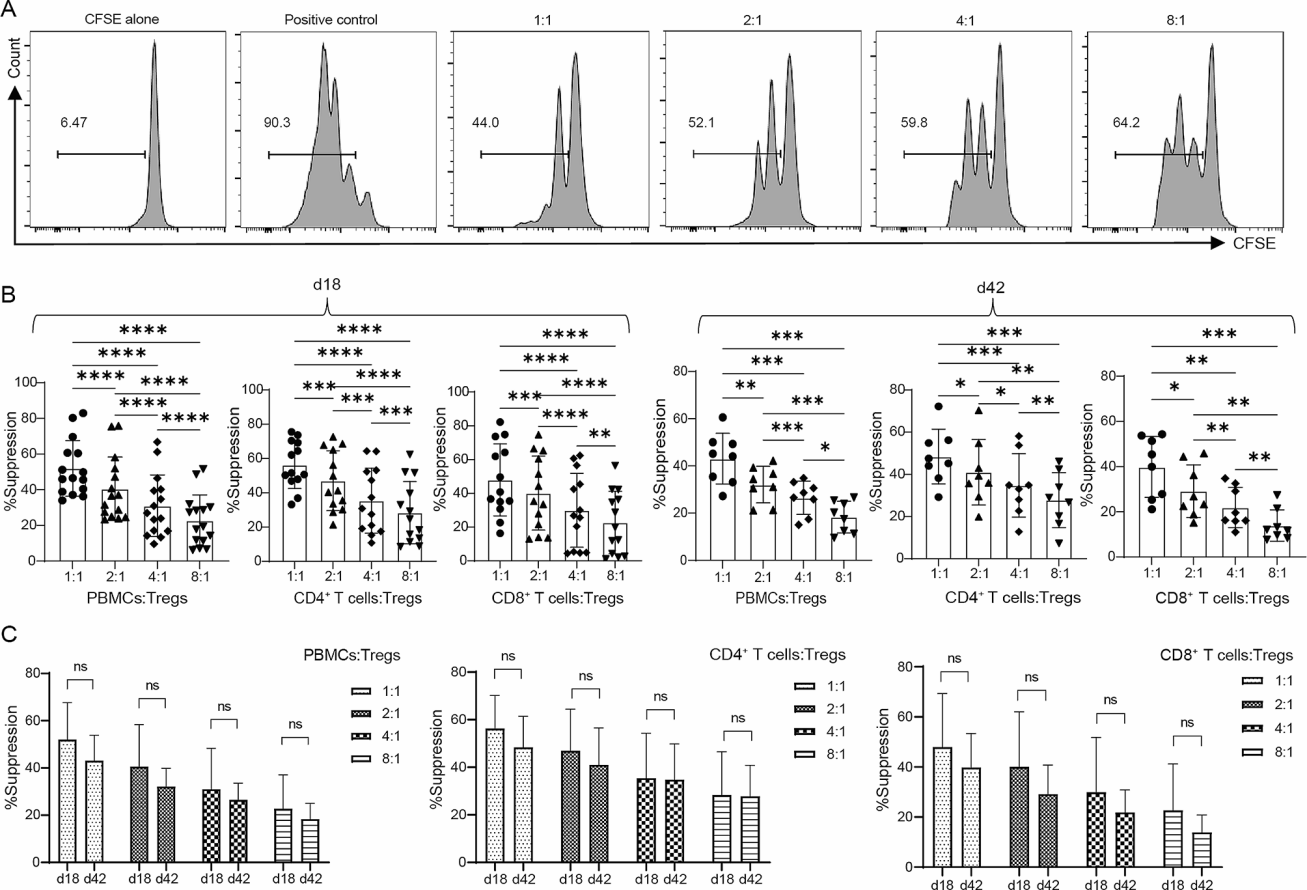
Suppressive function of UCB-Tregs expanded 18 days and 42 days. (A) Representative histograms of cell proliferation assay. (B) CFSE-labeled PBMCs were cocultured with UCB-Tregs by adding anti-CD3/28 beads at the indicated PBMC:Treg ratios of 1:1, 1:2, 1:4, and 1:8. CFSE alone: CFSE-labeled PBMCs without anti-CD3/28 beads. Positive control was CFSE-labeled PBMCs with anti-CD3/28 beads but without Tregs. Proliferation was determined via CFSE dilution on day 3; measurements were performed in duplicates. The proliferation of PBMCs, CD4+ T cells, and CD8+ T cells was directly measured via flow cytometry or after labeling with fluorescently conjugated anti-CD4 and anti-CD8 antibodies to distinguish the populations. The suppressive capacity was significantly different among the different proportions of expanded Tregs. The figures on the left show the coculture results of UCB-Tregs expanded for 18 days and the figures on the right depict the co-culture results of UCB-Tregs expanded for 42 days. Data are presented as the mean ± SEM. One-way ANOVA, followed by Tukey’s post hoc test was used; *p < 0.05, **p < 0.01, ***p < 0.001, ****p < 0.0001, n = 8–15. (C) No significant differences were observed in the suppressive function of d18 and d42 UCB-Tregs against PBMCs, CD4+ T cells, and CD8+ T cells. On the left is the coculture results of PBMCs with UCB-Tregs, in the middle is the coculture results of CD4+ T cells with UCB-Tregs, and on the right is the coculture results of CD8+ T cells with UCB-Tregs. Two independent sample t-test or Mann-Whitney test was used; n = 8–15

PBMCs were stained with CFSE while UCB-Tregs were not labeled before coculture. When analyzing the coculture data, CFSE, which identified PBMCs in the coculture system, was then used to analyze the proliferation of these cells. The majority of PBMCs or CD4^+^ T cells or CD8^+^ T cells stimulated with anti-CD3/CD28 beads proliferated; however, they did not proliferate significantly without anti-CD3/CD28 beads, as shown in Fig. 3A. More importantly, the proliferation was inhibited when these cells were cocultured with UCB-Tregs expanded for 18 or 42 days in vitro and the inhibition was proportional to the ratio of UCB-Tregs to other cells (Fig. 3B). The inhibition rate of UCB-Tregs to PBMCs or CD4^+^ T cells or CD8^+^ T cells (ratio 1:1) at 18 days was 52.00 ± 15.65%, 56.32 ± 13.83%, and 47.99 ± 21.34%, respectively. Furthermore, the suppressive function of UCB-Tregs at 42 days stimulated up to five times showed that the inhibition rate of UCB-Tregs to PBMCs or CD4^+^ T cells or CD8^+^ T cells (ratio 1:1) was 43.10 ± 10.74%, 48.46 ± 12.94%, 39.86 ± 13.48%, respectively, which were similar to the results of UCB-Tregs obtained at 18 days (Fig. 3C).

### Relationships between the suppressive function and clinical data of the subjects

**Fig. 4:**
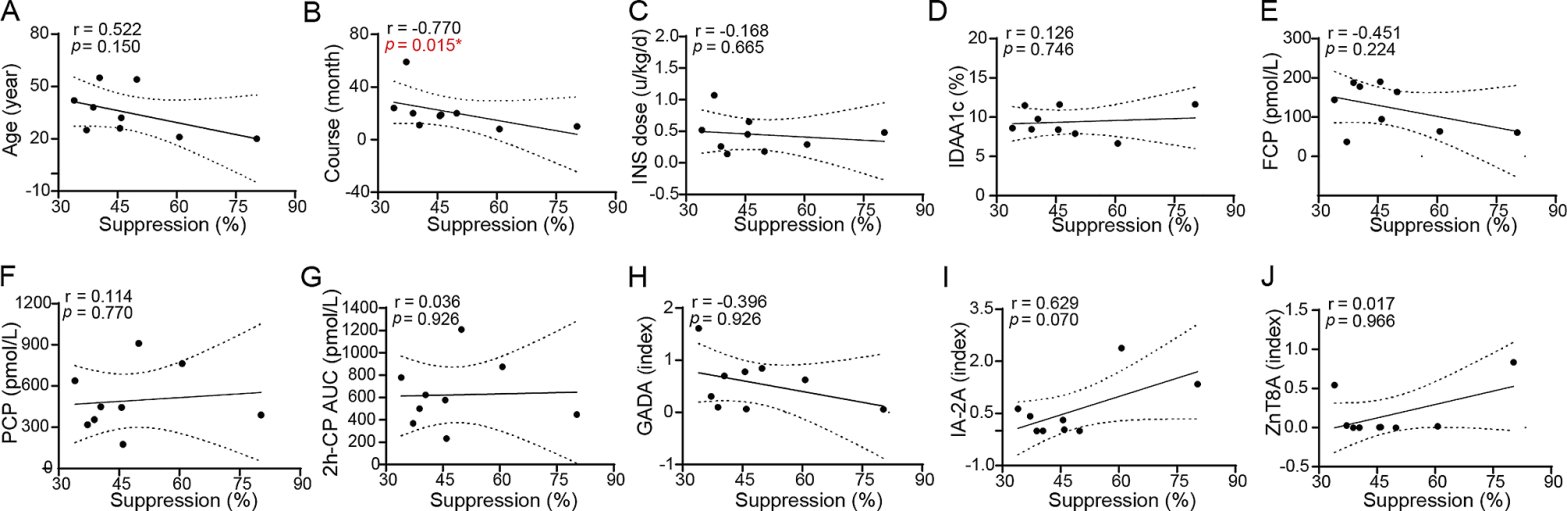
Relationships between suppression rate and clinical data of the subjects. Correlations between the suppression rate and age (A), course (B), INS dose (C), IDAA1c (D), FCP (E), 2-h PCP (F), 2-h CP-AUC (G), GADA (H), IA-2 A (I), and ZnT8A (J), *p < 0.05

The correlation between the clinical characteristics of patients and the suppression rate of UCB-Tregs to PBMCs at a 1:1 ratio was analyzed to investigate the factors that influence the ability of UCB-Tregs to inhibit the proliferation of target cells. The suppression rate of 18-day UCB-Tregs to PBMCs at a 1:1 ratio was negatively correlated with the course of diabetes (*r* = -0.770, *p* = 0.015) but not with age, daily insulin dose (INS dose), insulin dose-adjusted A1c (IDAA1c), FCP, 2-h PCP, 2-h C-peptide AUC, GADA, IA-2 A, and ZnT8A. Details are shown in Fig. 4A–J.

### Ability of the suppression rate of UCB-Tregs expanded for 18 days to predict patient condition during the 12-month follow-up period

**Fig. 5:**
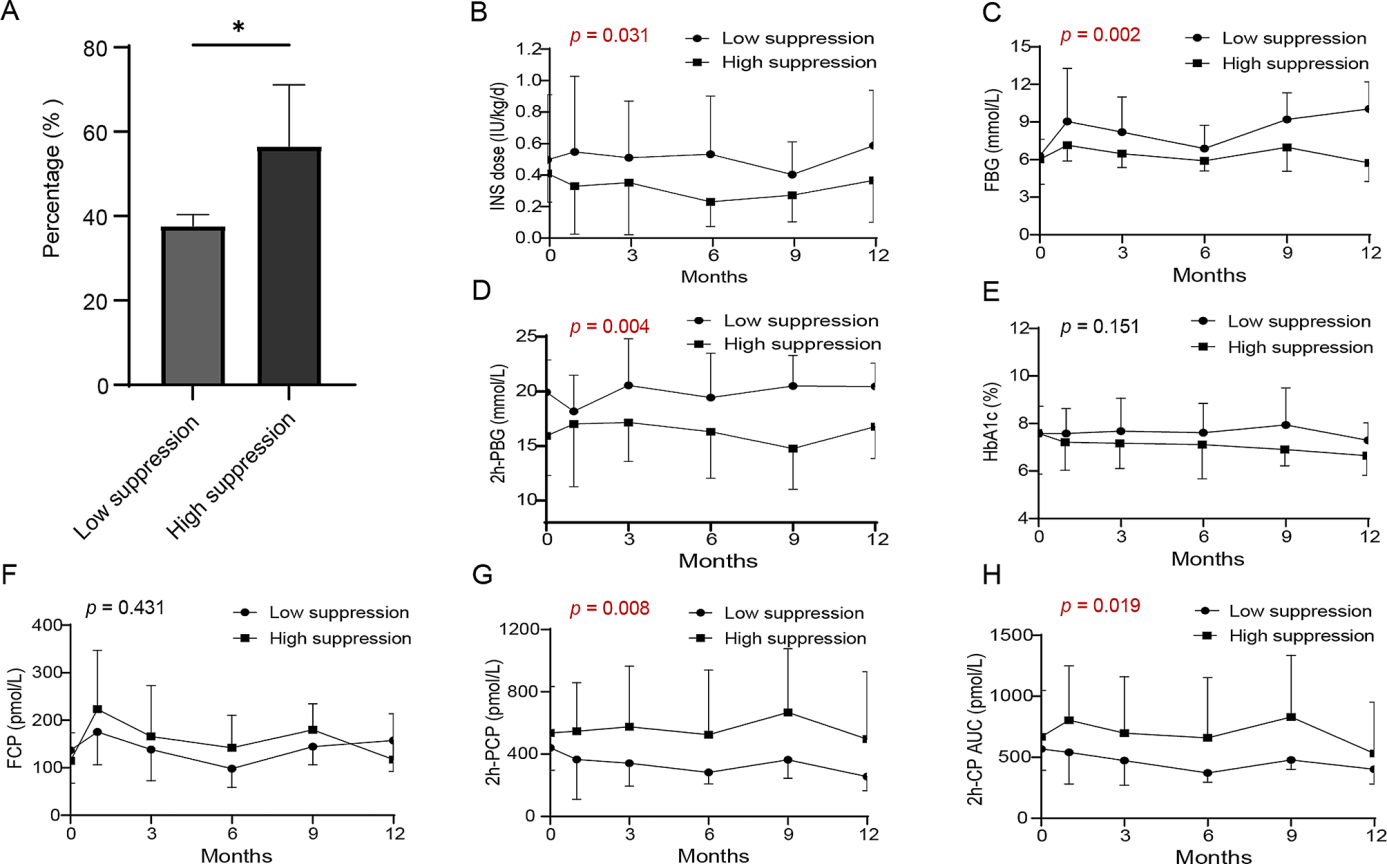
Ability of the suppression rate to predict the condition of patients during the 12-month follow-up. (A) Patients were divided into two groups (low- and high-suppression group) according to the median of the suppression rate. Changes in INS dose (B), FBG (C), 2-h PBG (D), HbA1c (E), FCP (F), 2-h PCP (G), and 2-h C-peptide AUC (H) in the two groups during the 12-month follow-up. Compared using GEE, n = 4 (low suppression) and 5 (high suppression); p values are shown in the figure

Patients were divided into low-suppression group and high-suppression group according to the median suppression rate of the UCB-Tregs to their PBMCs at a 1:1 ratio and we investigated whether the suppression rate could predict the treatment effect (Fig. 5A; Table 1). The baseline characteristics of the two groups are shown in Table 2. Demographic and baseline clinical characteristics were comparable between the two groups.

The high-suppression group had a lower INS dose (*p* = 0.031), lower FBG (*p* = 0.002), lower 2-h PBG (*p* = 0.004), higher 2-h PCP (*p* = 0.008), and higher 2-h C-peptide AUC (*p* = 0.019) (Fig. 5B–H) than the low-suppression group during the 12-month follow-up period.

**Table 1:** Suppression rate of each study test in the two groups

Group	Index	%Suppression (PBMCs:Tregs = 1:1)	%Suppression (PBMCs:Tregs = 2:1)	%Suppression (PBMCs:Tregs = 4:1)	%Suppression (PBMCs:Tregs = 8:1)
Low suppression	Mean	37.62	25.31	16.02	10.90
	SD	2.75	1.61	5.70	3.41
High suppression	Mean	56.48	47.46	39.79	31.22
	SD	14.64	16.22	15.78	10.78

**Table 2:** Baseline characteristics of the study subjects

	Low suppression group	High suppression group	p value
Gender			
Male	3/4	1/5	0.2060
Female	1/4	4/5	
Age (years)	40.00 ± 12.36	30.60 ± 13.92	0.3264
Diagnosis			
LADA	2/4	2/5	1.0000
T1D	2/4	3/5	
Course (months)	28.50 ± 21.05	15.00 ± 5.57	0.2052
FBG (mmol/L)	6.29 ± 1.34	6.02 ± 1.98	0.8221
2 h-PBG (mmol/L)	19.93 ± 2.95	15.92 ± 3.61	0.1178
FCP (pmol/L)	136.70 ± 69.05	114.70 ± 59.12	0.6221
2 h-PCP (pmol/L)	440.20 ± 143.30	536.60 ± 296.80	0.5733
2 h-CP AUC (pmol/L)	567.60 ± 175.10	668.00 ± 380.80	0.6440
HbA1c (%)	7.58 ± 1.15	7.60 ± 1.73	0.9825
INS dose (IU/kg/d)	0.50 ± 0.41	0.41 ± 0.18	0.6801

## Discussion

Herein, we described a method of multiple UCB-Treg stimulation rounds in the presence of rapamycin, resulting in a large-scale expansion of UCB-Tregs with a stable phenotype and inhibitory function. The in vitro suppression rate of UCB-Tregs cocultured with PBMCs was correlated with the disease course of patients and could predict the change in clinical indicators after 12 months follow-up. These results suggest that patients with a shorter duration of diabetes and higher suppression rate from in vitro coculture assay would experience more clinical benefits when transfused with ex vivo expanded polyclonal UCB-Tregs.

The potential of polyclonal Tregs to treat various diseases has been demonstrated earlier [24, 35, 39, 40]. Some studies have shown that antigen-specific Tregs are superior to polyclonal peripheral Tregs in controlling pancreatic autoimmunity [9, 41]. Whereas, only a small number of antigen-specific Tregs associated with a given disease are found in the peripheral blood, with most antigen-specific Tregs present in the tissues, making their isolation and the identification of their cognate antigens difficult. These obstacles inspired the design of strategies to target T cell receptors (TCRs) and chimeric antigen receptors (CARs) to engineer antigen-specific Tregs [42]. Of note, UCB can serve as an excellent source for producing functional antigen-specific Tregs for immunotherapy [43].

We used magnetic sorting devices to purify CD4^+^CD25^+^CD127^−^ Tregs from UCB. The purity of freshly sorted Tregs was evaluated after in vitro expansion for 18 days, and the results obtained were superior to those of previous studies [35, 44]. The present study approach was more quickly and cost-effectively compared with flow cytometry-based purification [45]. Although no perfect markers for Tregs exist, Liu et al. [46] and Seddiki et al. [47] have shown that the CD25^high^CD127^low^ population of CD4^+^ T cells is 90% correlated with Foxp3 expression, thereby enabling a better identification of true Tregs from effector (CD4^+^CD25^+^CD127^high^) cells. In UCB, staining with anti-CD127 revealed that the CD25^+^ subset was not completely homogeneous but consisted of a mixture of CD25^+^CD127^lo^ and CD25^+^CD127^high^ cells [47]. Therefore, the addition of CD127 enabled the sorting of pure Tregs [48]. Moreover, other cells that could have increased the complexity of the amplification system were excluded. The stable large-scale amplification of UCB-Tregs is conducive to obtaining ready-made Treg products for multiple treatment cycles in patients. Indeed, an expansion of 123 ± 60-fold at 18 days and 5,981 ± 5,721-fold at 42 days was obtained in this study. The amplification efficiency of Tregs from different UCB units varied. Thus, this aspect should be further investigated.

Previous research [49] revealed that repetitive in vitro stimulation decreased Foxp3 expression in partial Tregs. Moreover, their inhibitory capacity was lost and many cells died after more than two expansion cycles [30]. Here, we demonstrated that the expression of Foxp3 did not decrease after up to five repetitive stimulations. Our results also suggested that the expression of CTLA-4 and GITR, two important immunosuppressive molecules of Tregs, increased after expansion and remained relatively high after long-term expansion, similar to the results reported by Hippen et al. [50]. UCB-Tregs restimulated for five cycles in rapamycin achieved up to 22,310-fold expansion over a 42-day period and the Treg percentage remained stable without affecting the ability to inhibit the proliferation of PBMCs, CD4^+^ T cells, or CD8^+^ T cells.

To modulate Tregs into an effective therapeutic option, a better understanding of their various functions and heterogeneous characteristics is needed. Based on their developmental origins, Tregs can be classified into two major groups: tTregs and pTregs [51]. tTregs develop in the thymus and express the transcription factor Foxp3. pTregs with or without Foxp3 expression originate from naïve T cells that migrate to the peripheral circulation and are activated by exogenous antigens and environmental signals. pTregs without Foxp3 expression include interleukin-10-producing type 1 regulatory T (Tr1) cells, transforming growth factor-β-producing T helper 3 (Th3) cells, and others [52]. In vitro, Tregs induced from naïve T cells are called induced Tregs (iTregs) [52]. Of note, tTregs have a more stable epigenetic program [53] and are rather resistant to reversion into pathogenic CD4^+^ T cells compared with pTregs derived from peripheral naïve CD4^+^ T cells or iTregs induced in vitro from naive CD4^+^ T cells [54, 55].

To our knowledge, this is the first study to analyze the factors of patients with autoimmune diabetes that influence the in vitro suppressive function of Tregs. The results show that the suppression rate is negatively correlated with the duration of the patients’ disease, suggesting that the shorter disease duration is associated with better response to treatment when selecting patients with autoimmune diabetes for adoptive Treg transfer treatment. Marek-Trzonkowska et al. [23] conducted a clinical trial on autologous ex vivo expanded Tregs for T1D, and the results similarly indicated that the best responders to the treatment were children with a short disease duration at the time of the enrollment. A clinical trial of teplizumab therapy for T1D suggested that patients with newly diagnosed T1D (i.e., within 100 days from diagnosis) and who were younger were more likely to benefit from this immunotherapy [56]. Our findings did not show a correlation between age and treatment response, but an association between shorter disease duration and better treatment response was observed. This suggests that newly diagnosed patients with more residual islet function are more likely to benefit from UCB-Treg therapy. There are several potential reasons for this observation, including the evolution of the immune response over the disease course and the effect of prolonged damage from which beta cells cannot be recovered. In addition, the HLA haplotype, disease activity, glycemic severity, immune biomarkers, specific islet autoantibody (AAb) positivity, and environmental exposures [57] may help identify these potential responders, which requires further exploration.

Whether in vitro inhibition assays can predict the effect of treatment in vivo has not been shown in previous studies. Our results showed that the high-suppression group had lower INS dose, lower blood glucose, and higher C-peptide levels than the low-suppression group. The in vitro inhibition assay predicts the in vivo response of the treatment, providing a possibility for a more precise selection of appropriate patients. Similar to our study, Rosenzwajg et al. [58] classified patients into Treg high-responders and Treg low-responders based on the proportional increase in Treg response from the baseline on day 5. Higher C-peptide AUC level was observed in Treg high-responders at 351 and 436 days of follow-up. Another research showed that a shift in naïve and memory Tregs was influenced by inflammation associated with long-lasting T1D [59]. Therefore, the level of inflammatory factors in vivo might affect the efficacy of UCB-Treg therapy, which should be further explored.

Our study has some limitations. First, the sample size was small. Second, it was a single institution study. Third, it is slightly inconvenient to use in vitro suppression assay to predict treatment response after UCB-Tregs have been expanded for several days. However, there is a lack of research on the prediction of treatment effects and our study provides new insights in this regard. We acknowledge these limitations and have planned to conduct a multicentric study with a larger patient sample size to produce more representative results. In addition, we will continue to improve our method of producing UCB-Tregs; explore more possible predictors with regard to UCB-Tregs, patients themselves, and the interaction between UCB-Tregs and patients’ immune cells; and move toward the goal of precision medicine.

## Conclusion

Multiply restimulated UCB-Tregs expanded on a large scale without altering their classical phenotypic features and inhibitory functions, thereby providing an off-the-shelf Treg production method. Furthermore, our work demonstrated that the suppressive function of UCB-Tregs in vitro was correlated with the disease duration of UCB cell donors. Indeed, the shorter the disease duration, the stronger the suppressive function of Tregs on the immune cells. Additionally, our work revealed the possibility of predicting in vivo therapeutic effects via in vitro inhibition assay, facilitating the selection of patients who are more likely to respond to treatment. This may help advance clinical practice toward the goal of individualized and precision treatment. However, the study findings need to be interpreted with caution and further validated via well-designed prospective studies in the future owing to the potential confounding factors and small sample size in this study.

## Data Availability

Data is contained within the article. The data presented in this study are available from the corresponding author on reasonable request.
