# Correction: Multiply restimulated human cord blood-derived Tregs maintain stabilized phenotype and suppressive function and predict their therapeutic effects on autoimmune diabetes

**DOI:** 10.1186/s13098-024-01372-2

**Published:** 2024-07-04

**Authors:** Yuanjie Bi, Ran Kong, Yani Peng, Donghua Cai, Yu Zhang, Fan Yang, Xia Li, Wen Deng, Fang Liu, Binbin He, Chuqing Cao, Chao Deng, Xiaohan Tang, Li Fan, Haibo Yu, Zhiguang Zhou

**Affiliations:** 1 National Clinical Research Center for Metabolic Diseases, Key Laboratory of Diabetes Immunology, Ministry of Education, Hunan Engineering Research Center of Cell Therapy for Diabetes, and Department of Metabolism and Endocrinology, The Second Xiangya Hospital of Central South University, Changsha, China; 2 Department of Obstetrics and Gynecology, The Second Xiangya Hospital of Central South University, Changsha, China

**Correction: Diabetology & Metabolic Syndrome (2024) 16 :71**

10.1186/s13098-024-01277-0

Following publication of the original article [1], the author reported that the corresponding author’s symbol has been missed for one of the authors in the author group. Haibo Yu1† is one of the corresponding author and it is presented correctly in this correction article.

The original article has been corrected.

